# Efficacy and safety for Apatinib treatment in advanced gastric cancer: a real world study

**DOI:** 10.1038/s41598-017-13192-8

**Published:** 2017-10-16

**Authors:** Yong Zhang, Chun Han, Juan Li, Li Zhang, Lijie Wang, Sisi Ye, Yi Hu, Li Bai

**Affiliations:** Oncology Department, Chinese PLA General Hospital, Beijing, China

## Abstract

Apatinib has been proved to be effective and safe among patients in gastric cancer in Phase II and III Trials. We aimed to evaluate its efficacy and safety in real world practice, and to explore factors associated with efficacy. Between January 2015 and February 2017, totally 36 patients with advanced gastric adenocarcinoma or adenocarcinoma of gastroesophageal junction (GEJ) were enrolled and followed up retrospectively after failing at least two lines of systemic therapy. The mPFS was 2.65 months (95%CI 1.66–3.54), and mOS was 5.8 months (95%CI 4.77–6.83). Two patients achieved partial response, and nineteen achieved stable disease. The disease control rate (DCR) was 58.3%, and objective response rate (ORR) was 5.6%. Common grade adverse events were hypertension (38.9%), proteinuria (36.1%), and neutropenia (33.3%). And the most common adverse events over grade 3 were hand-foot syndrome (8.3%), anemia (5.6%), and diarrhea (5.6%). No treatment-related death was documented during the drug administration. Exploratory analyses indicated patients treated with antiangiogenic therapy previously were more likely to benefit from apatinib.

## Introduction

Gastric cancer (GC), including cancer in gastroesophageal junction (GEJ), is a common digestive system neoplasm, which is the third leading cause of cancer related death worldwide^1^. Surgery is considered to be the only radical treatment for early diseases, however, recurrence incidence remains high in patients after the multidisciplinary approach involving radical resection and perioperative or adjuvant treatment, not mention to that approximately 80% of patients with locally advanced or metastatic GC can barely receive benefit from gastrostomy^2^. Chemotherapy typically based on platinum or taxanes, has prolonged the average overall survival time to nearly 12 months, showing a limited effect^3^. At present, new approaches are focusing on molecularly driven therapies.

Angiogenesis is one of the most important mechanisms for the emergence and development of malignant tumors. Angiogenesis contributes to the processes of tumor proliferation, metastasis, and migration, acting as nutrient supply for cancer cells^4^. Therefore, antiangiogenic therapy has become a prior choice to conflict with cancers, and a few angiogenesis inhibitors have shown efficacy in lung, breast, and colon cancers^5,6^. Although in gastric cancer the evidence is still inconclusive, antiangiogenic treatment is also considered a promising therapy^7–10^.

Apatinib is a small-molecule tyrosine kinase inhibitor (TKI) that highly selectively binds to and strongly inhibits vascular endothelial growth factor receptor 2 (VEGFR-2). Previous phase II and III clinical trials has shown that its efficacy and safety in patients with chemotherapy-refractory advanced or metastatic gastric carcinoma when compared with placebo^11,12^. And in these trials, the therapeutic effect of apatinib on overall survival was mainly derived from prolonged progression-free survival^13^. However, the treatment of apatinib in the real world is still unclear.

Therefore, we carried out this observational study to give more clinical evidence of the treatment of apatinib in patients with gastric cancer and cancer of GEJ in the real world.

## Results

### Patients and tumor characteristics

A total of 36 patients with advanced gastric adenocarcinoma or adenocarcinoma of the gastroesophageal junction (GEJ) who had progressed or relapsed after undergoing at least two lines of systemic therapy in Oncology Department of the Chinese PLA General Hospital (PLAGH) between January 2015 and February 2017 were included. The median age of the patients was 58 years old, ranging from 38 to 75, and 69.4% were male. All patients were histologically confirmed adenocarcinoma, with or without some other components, which included mucinous adenocarcinoma or signet ring cell carcinoma. There were 8 patients with Her2 positive status (22.2%), 12 patients with Her2 negative status (33.3%), except that 16 patients didn’t carry out this test due to personal preference, insufficient biopsy specimen or financial difficulties (44.4%). All patients had advanced or metastatic disease and the most common metastatic sites were liver (55.6%), distant lymph nodes (50.0%) and peritoneum (22.2%). Thirty-one patients had an ECOG performance status of 0/1 (86.1%), and 5 patients’ ECOG PS was 2/3 (13.9%).

All patients had received previous treatment, including but not limited to gastrostomy, chemotherapy, radiotherapy and targeted therapy. Twenty-four patients (66.7%) hadn’t undergone any surgery while 10 patients (27.8%) had radical surgery and 2 patients (5.6%) had palliative surgery. Five patients (13.9%) had radiotherapy due to positive surgical margin. Most patients had received doublet or triplet chemotherapy in the first and second line therapy and the most important chemotherapeutics were Platinum, Taxanes and Fluorouracil. All 8 patients with positive Her2 status had received anti-Her2 therapy in the first or second line according to guidelines. Nine patients (25%) had undergone antiangiogenic therapy in first or second line therapy involving bevacizumab and apatinib. In these 9 patients’ previous treatment, bevacizumab was combined with therapy including XELOX, DOF and Everolimus, while apatinib was applied alone.

Complete clinical and pathologic characteristics at the initiation of apatinib therapy are shown in Table 1, and the previous treatment details are provided in Tables 2 and 3.

**Table 1:** Patients and tumor characteristics (N = 36).

Characteristics	No.	%
Total	36	100.0%
Age (years)		
Median	58	
Range	38–75	
Gender		
Male	25	69.4%
Female	11	30.6%
Primary lesion		
Gastric	25	69.4%
Gastroesophageal junction	11	30.6%
Histology		
Adenocarcinoma	31	86.1%
Adenocarcinoma with other components	4	11.1%
Mucinous adenocarcinoma	1	2.8%
Differentiation		
Poorly	14	38.9%
Moderately	17	47.2%
Highly	2	5.6%
Unknown	3	8.3%
Her2 status		
Negative	12	33.3%
Positive	8	22.2%
Unknown	16	44.4%
Metastasis at Stage IV diagnosis		
Liver	20	55.6%
Lung(s)	4	11.1%
Peritoneum	8	22.2%
Distant lymph node	18	50.0%
No. of metastatic sites		
≤2	6	16.7%
>2	30	83.3%
ECOG PS		
0	7	19.4%
1	24	66.7%
2	4	11.1%
3	1	2.8%

(Adenocarcinoma with other components included mucinous adenocarcinoma or signet ring cell carcinoma. Her2 negative status included IHC score 0, 1, 2 without gene amplification in FISH, while positive status included IHC score 2 with gene amplification in FISH. Distant lymph nodes included Supraclavicular lymph nodes, Posterior peritoneum lymph nodes, and other lymph nodes. One patient could have several metastatic lesions and there were some other infrequent metastatic site, such as osseous, adrenal, ovarian metastasis).

**Table 2:** Previous treatment (N = 36).

Treatment	No.	%
Prior gastrostomy		
Radical surgery	10	27.8%
Palliative surgery	2	5.6%
No surgery	24	66.7%
Prior radiotherapy		
Yes	5	13.9%
No	31	86.1%
Prior chemotherapy		
2 lines	25	69.4%
3 lines	8	22.2%
4 lines	2	5.6%
5 lines	1	2.8%
Prior targeted therapy		
Antiangiogenic therapy	9	25.0%
Anti-Her2 therapy	8	22.2%
No targeted therapy	19	52.8%

(Antiangiogenic therapy included bevacizumab or apatinib therapy).

**Table 3:** Previous antiangiogenic treatment details (N = 9).

Patient	Study line	Antiangiogenic therapy before study	Apatinib therapy in study line	PFS
1	3	B + XELOX	A	1.2
2	3	B + DOF	A	3.6
3	3	B + D	A + S	3
4	4	B + everolimus	A + Iri	2.2
5	3	A	A + S	3.6
6	3	A	A + Iri	3.4
7	3	A	A + X	8.6
8	3	A	A + D	5.7
9	4	A	A + O	1.8

(A: apatinib; B: bevacizumab; XELOX: oxaliplatin and capecitabine; DOF: docetaxel, oxaliplatin, and fluorouracil; D: docetaxel; S: S-1; Iri: Irinotecan; X: capecitabine; O: oxaliplatin).

### Treatment administration

Twenty-one patients (58.3%) had started the apatinib therapy from the dosage of 500 mg, and 13 (36.1%) from 250 mg, and 2 (5.6%) from a dosage that was higher than 500 mg. Five patients (13.9%) had decreased their initial dosage for the reason of intolerable toxicity. Twelve patients (38.9%) had received concomitant chemotherapy on physicians’ choices, including 2 patients using doublet chemotherapy. Combined chemotherapy involved Fluorouracil (8 patients, 22.2%), Platinum (3 patients), Irinotecan (2 patients, 5.6%) and temozolomide (1 patient). Two patients combined with other treatment involving radiotherapy (1 patient) and ablation (1 patient). At the cutoff time (April 20th, 2017), all these 36 patients had discontinued apatinib therapy on account of disease progression.

### Efficacy

**Figure 1:**
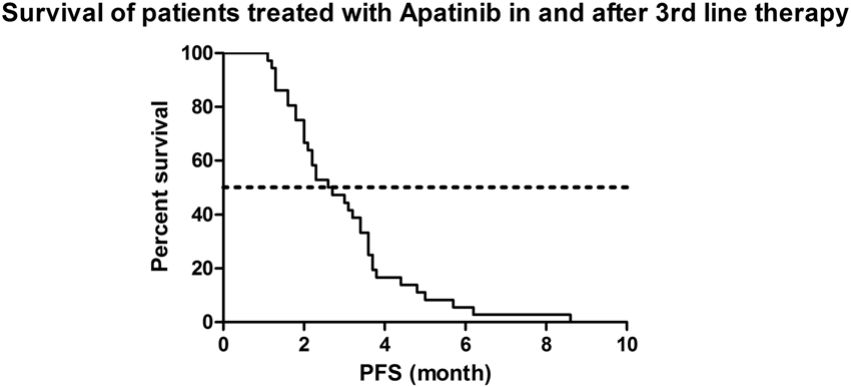
Kaplan-Meier estimates of progression-free survival of patients treated with Apatinib in and after 3rd line therapy. (N = 36, median PFS = 2.65 mo, 95%CI 1.66–3.54).

**Figure 2:**
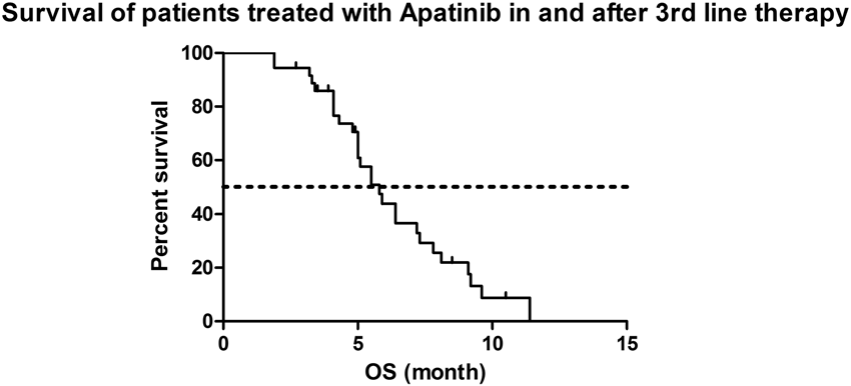
Kaplan-Meier estimates of overall survival of patients treated with Apatinib in and after 3rd line therapy. (N = 36, median OS = 5.8 mo, 95%CI 4.77–6.83).

At the time of analysis, all patients had progressed from apatinib therapy and 28 patients (77.8%) had died mainly because of tumor progression. The median PFS was 2.65 months (95%CI 1.66–3.54), and the median OS was 5.8 months (95%CI 4.77–6.83). The Kaplan-Meier curves of PFS and OS are shown in Figs 1 and 2.

All the 36 patients had been evaluated by imageological examination. Two patients (5.56%) achieved PR, 19 patients (52.78%) had SD, and 15 patients (41.67) were reported as PD after the apatinib therapy. These resulted in an ORR of 5.56% and a DCR of 58.33%.

As shown in Table 4, seven factors including age group, prior gastrostomy, liver metastasis, peritoneal metastasis, previous targeted therapy (anti-Her2 and antiangiogenic) and combination therapy, were brought into our exploratory analysis in consideration of clinical practice and previous study. Cox regression model showed a significant association between apatinib therapy PFS and prior antiangiogenic therapy (adjusted HR = 2.935, 95%CI 1.047–8.229, P = 0.041). Besides that, factors considered as potential markers associated with the efficiency like liver metastasis, peritoneal metastasis and number of metastasis didn’t make any differences in our study, probably because of small sample size. Data of univariate analysis and multivariate analysis were shown in Table 4.

**Table 4:** Exploratory analysis of factors to predict PFS of Apatinib.

	n	mPFS (m)	univariate analysis			multivariate analysis		
			P	HR	95%CI	P	Adjusted HR	95%CI
Age group								
<median = 58	19	2.3	0.642	1.174	0.597–2.308	0.375	1.452	0.673–3.310
≥ median = 58	17	3						
Prior gastrostomy								
Yes	12	2	0.808	0.914	0.444–1.882	0.216	0.554	0.217–1.412
No	24	3						
Liver metastasis								
Yes	20	2.3	0.059	0.500	0.244–1.027	0.071	0.475	0.212–1.065
No	16	3.6						
Peritoneal metastasis								
Yes	8	2	0.122	0.522	0.229–1.190	0.213	0.561	0.226–1.392
No	28	3						
Prior anti-Her2 therapy								
Yes	12	3.4	0.538	1.253	0.611–2.569	0.061	2.487	0.959–6.449
No	24	2.2						
Prior antiangiogenic therapy								
Yes	9	3.4	0.266	1.577	0.706–3.520	0.041	2.935	1.047–8.229
No	27	2.3						
Combination therapy								
Apatinib only	22	2.3	0.503	0.790	0.396–1.575	0.703	1.166	0.531–2.559
Combined with other therapy	14	3						

(Antiangiogenic therapy included bevacizumab or apatinib therapy).

### Safety

All 36 patients were included in safety analysis. Toxicities were generally well tolerated. Dose modifications resulting from toxicity occurred in 5 patients. The reasons for dosage decrement were hypertension, proteinuria, leukopenia, and thrombocytopenia.

The most common adverse events included hypertension (38.9%), proteinuria (36.1%), nausea and vomiting (36.1%), and leukopenia (33.3%). Grade 3 to 4 AEs with the incidences of 5% or greater were hand-foot syndrome (8.3%), anemia (5.6%) and diarrhea (5.6%). Only one patient occurred grade 4 AE, hand-foot syndrome.

These findings are consistent with those from previous clinical trials. No treatment-related death was documented during the drug administration. All adverse events are listed in Table 5.

**Table 5:** Adverse events (N = 36).

	G1	G2	G3	G4	Grade ≥ 1	%	Grade ≥ 3	%
Hypertension	10	3	1	0	14	38.9%	1	2.8%
Proteinuria	10	2	1	0	13	36.1%	1	2.8%
Hand-foot syndrome	1	3	2	1	7	19.4%	3	8.3%
Leukopenia	8	3	1	0	12	33.3%	1	2.8%
Neutropenia	4	4	0	0	8	22.2%	0	0.0%
Anemia	3	1	2	0	6	16.7%	2	5.6%
Thrombocytopenia	4	2	1	0	7	19.4%	1	2.8%
Elevated transaminase	3	4	1	0	8	22.2%	1	2.8%
Hyperbilirubinemia	5	3	0	0	8	22.2%	0	0.0%
Bleeding	2	0	0	0	2	5.6%	0	0.0%
Nausea and Vomiting	6	7	0	0	13	36.1%	0	0.0%
Diarrhea	5	2	2	0	9	25.0%	2	5.6%
Fatigue	5	2	0	0	7	19.4%	0	0.0%

## Discussion

Fluoropyrimidine-based, platinum-based and taxanes-based chemotherapy, whether two-drug regimens or three-drug regimens, have been widely used in routine first line treatment for locally advanced or metastatic gastric cancer^14–17^. NCCN guidelines also recommend six preferred regimens for second line therapy. However, only a few studies assessed third-line choices for gastric cancer. Sakura Iizumi reported taxane monotherapy including paclitaxel and nab-paclitaxel for GC in 3rd line, showing efficacy (mPFS = 2.0 mo, mOS = 4.5 mo) and safety (G3/4 AE ratio = 33.3%) in a multicenter retrospective study^18^. Takashi Nishimura reported Irinotecan monotherapy efficacy (mPFS = 2.3 mo, mOS = 4.0 mo, ORR = 3%, DCR = 22%) and safety (G3/4 AEs: neutropenia 27%, febrile neutropenia 12%, anorexia 12%)^19^. Eun Joo Kang reported FOLFIRI efficacy (mPFS = 2.1 mo, mOS = 5.6 mo, ORR = 9.6%) and safety (G3/4 AE: myelosuppression 36.7%)^20^. All these chemotherapies have attempted to achieve low toxicity and good efficacy, which could be optimized and improved for further.

Our study is a real-world observation of the efficacy and safety of the apatinib therapy for patients with advanced gastric cancer or advanced GEJ cancer. In this study of the third or more line treatment for gastric cancer, apatinib therapy led to a median PFS of 2.65 months (95%CI 1.66–3.54), a median OS of 5.8 months (95%CI 4.77–6.83), an ORR of 5.56% and a DCR of 58.33%. Common adverse events were hypertension (38.9%), proteinuria (36.1%), and neutropenia (33.3%). And the most common adverse events over grade 3 were hand-foot syndrome (8.3%), anemia (5.6%), and diarrhea (5.6%). As shown in Table 6, our apatinib therapy seems to be nearly efficient and safe compared with phase II and III trials^11,12^. However, this is a real world study, an observational study, which mingled with some complicated factors. There is still some dissimilitude between them. On one hand, patients’ performance status before apatinib in this study was much worse than in clinic trails. Five patients ECOG PS was 2/3 (13.9%) in our study, while all patients in previous clinic trails scored 0 or 1. In our study, 83.3% of the patients had more than two metastatic lesions, while the corresponding rate in phase III trial was 21%. Only 33.3% the patients in present study had received surgery, involving radical and palliative surgery at the same time, while the rate in phase III trial was 69.3%. Besides that, patients in our study were heavily pretreated in consideration of the three patients who had received over four lines chemotherapy. These discrepancies illustrate patients in real world perform worse and have more visceral metastases and higher tumor burden, and highlight the gap in the baseline of apatinib treatments between the randomized controlled trials and real-world treatment. On the other hand, dose titration was more flexibly in our study although initial apatinib dosage of 500 mg once daily in our study was situated between the two clinic trials, which could be increased to 750 mg or decreased to 250 mg. Combination with other therapy was also allowed according to their actual performance status in our study, which wasn’t covered in trials. We believed it was these rectifications in the treatment method that we had obtained similar efficacy results with previous trials, even if patients were heavily pretreated and performed worse. Moreover, these rectifications, especially dose up-regulation and combination chemotherapy, didn’t increase the incidence of adverse events, which meant a well-behaved tolerance of patients.

**Table 6:** Comparison with previous studies.

Parameters		PLAGH	Phase II Trial		Phase III Trial
			850 mg QD	425 mg BID	850 mg QD
No. of patients		36	47	46	176
Survival					
median OS (months)		5.8	4.83	4.27	6.5
	95% CI	4.77–6.83	4.03–5.97	3.83–4.77	4.8–7.6
median PFS (months)		2.65	3.67	3.2	2.6
	95% CI	1.66–3.54	2.17–6.80	2.37–4.53	2.0–2.9
Responses					
ORR		5.56%	6.38%	13.04%	2.84%
DCR		58.33%	51.06%	34.78%	42.05%
Adverse events					
Hypertension	Grade ≥ 1	38.90%	40.43%	39.13%	35.20%
	Grade ≥ 3	2.80%	8.51%	10.87%	4.50%
Proteinuria	Grade ≥ 1	36.10%	27.66%	34.78%	47.70%
	Grade ≥ 3	2.80%	2.13%	4.35%	2.30%
Hand-foot syndrome	Grade ≥ 1	19.40%	25.53%	45.65%	27.80%
	Grade ≥ 3	8.30%	4.26%	13.04%	8.50%

(Survival and response data in Phase II Trial were from intent-to-treat (ITT) patients. In Phase III Trial, survival data were from the full analysis set (FAS) patients, and response data were assessed by investigators).

Preclinical data demonstrated that vascular endothelial growth factor (VEGF) was continuously expressed during oncogenesis, tumor growth and metastasis, by facilitating tumor angiogenesis, and prolonged exposure to VEGF inhibitors could delay tumor growth and even maintain tumor regression^21–23^. Continuous angiogenic blockade strategy has been evaluated in the clinical settings and been proved to benefit patients with metastatic colorectal cancer^24–26^, but there is still no evidences directly supporting this concept in gastric cancer. In our exploratory analysis, multivariate analysis indicated prior antiangiogenic therapy was an independent factor associated with PFS of apatinib therapy. Nine patients had undergone antiangiogenic therapy in prior treatment, involving apatinib and bevacizumab. As shown in Table 3, four patients received bevacizumab combined with chemotherapy or targeted therapy regularly, and 5 received apatinib alone in second or third line before they were brought into our study. After disease progression from previous treatment, they continued antiangiogenic therapy with apatinib and benefited from it, whether a small-molecule TKI selectively targeting VEGFR-2 (apatinib) or a monoclonal antibody targeting VEGF ligand (bevacizumab) was used before. Bevacizumab as a continuous therapy had been discussed in the ML18147 trial and the BEBYP trial, in which bevacizumab was continued or reintroduced after the first progression of bevacizumab interruption, and bevacizumab plus chemotherapy significantly prolonged patients OS and PFS (OS in ML18147, B + C 11.2 mo vs. C 9.8 mo, HR = 0.81, 95%CI 0.69–0.94, P = 0.0062; PFS in BEBYP, B + C 6.8 mo vs. C 5.0 mo, HR = 0.70, 95%CI 0.52–0.95, P = 0.010)^24,27^. But this strategy still needs more exploration and discussion.

Antiangiogenic therapies, no matter monoclonal antibodies or tyrosine kinase inhibitors, were usually combined with chemotherapy, because of the poor efficiency used alone. Preclinical models demonstrated that sustained monoclonal antibody antiangiogenic treatment could create or remodel an environment suitable for normalization of the stable vascular endothelial cells, leading to increased tumor uptake of chemotherapy, which could be a possible explanation for the beneficial effect of this combination therapy^28,29^. However, the combined therapy strategy involving antiangiogenic and chemo therapy in gastric cancer has long been controversies. Most evidences were inclined to support the counterview. Monoclonal antibodies such as bevacizumab or ramucirumab, neither used alone nor combined with chemotherapy could significantly improve patients’ survival. For instance, in AVATAR study, ST03 study, REGARD study and RAINBOW study, addition of bevacizumab or ramucirumab didn’t show any advantages^8,30–32^, and the same results also occurred in TKIs, like orantinib, pazopanib and sorafenib^33–36^. Only two researches showed that monotherapy of antiangiogenic TKIs could significantly prolong the PFS as the primary endpoint, including apatinib (Phase III, in 3rd line, apatinib 2.6 mo vs. placebo 1.8 mo, HR = 0.44, 95%CI 0.54–0.94, P < 0.001) and regorafenib (Phase II, in 2nd or 3rd line, regorafenib 2.6 mo vs. placebo 0.9 mo, HR = 0.40, 95%CI 0.28–0.59, P < 0.001)^11,37^. In our study, fourteen patients added other systemic and local treatment, including chemotherapy, radiotherapy and ablation to apatinib therapy. However the combination didn’t significantly prolong patients’ PFS compared to apatinib, which was ascribed to differences in tumor biology and subsequent treatment between the first and second line settings, or limitations of study designs and patient selection. We also noticed the five patients in Table 3 who progressed from apatinib monotherapy but benefited from the combination of apatinib plus chemotherapy, and we attributed this to the enhancement of apatinib to conventional chemotherapy. In preclinical data, apatinib significantly increased the intracellular accumulation of rhodamine 123 and doxorubicin in cells by down-regulating the expression of P-glycoprotein (P-gp, ABCB1) or breast cancer resistance protein (BCRP, ABCG2), then reversed the multidrug resistance (MDR) and significantly enhanced the cytotoxicity substrate drugs^38,39^. Further studies remain needed.

Consequently, rigorous randomized controlled trials are needed to establish whether apatinib with an alternative chemotherapy regimen can benefit patients more than apatinib monotherapy, whether apatinib with or without an alternative chemotherapy regimen can benefit patients who have progressed after previous antiangiogenic therapy, or whether antiangiogenic therapy history can predict the advantaged population of apatinib therapy.

In spite of the inspiring improvement of PFS and DCR, apatinib has also exposed patients to its toxicity. It draws more and more attention of physicians and patients before considering the administration of apatinib. Hypertension, proteinuria and hand-foot syndrome are the most common AEs in antiangiogenic therapy^7,8,40^. In our present study, the safety profile was almost consistent with previous clinical trials, except that the incidence of certain AE hand-foot syndrome was a little lower^11,12^. Dealing with hand-foot syndrome has always been a tough thing. We attribute the lower incidence of hand-foot syndrome to prophylactic treatment, including moisturization for hand and foot, herbal medicine immersion and supplementation of multi-vitamin. Hypertension, proteinuria were treated in accordance with standard principles. In addition, hematologic toxicities related to apatinib in our study included leukopenia and neutropenia, while common non-hematologic toxicities involved elevated transaminase, hyperbilirubinemia, nausea and vomiting and diarrhea. Given that 66.7% of our patients not received any surgery, the incidence of bleeding events had not increased obviously compared to clinical trials. It indicates that apatinib does not increase risks associated to antiangiogenic therapy and can be tolerated by patients with heavy tumor burden of primary lesion. Besides that, a puzzle emerged in the present study, how to define the bleeding. For instance, should we categorize positive result of fecal occult blood as hemorrhage in digestive tract? On one side, primary lesion itself of advanced gastric cancer could lead a positive result, which might lead to an overestimated incidence. On the other side, in clinic work, fecal occult blood is usually not monitored as frequently as other laboratory examination such as blood routine examination. Only when patients noticed the red change of their fecal color, or when they experienced abdominal pain or diarrhea symptom, this test would be monitored regularly. Therefore, the incidence was highly likely to be underestimated. On the whole, from the observations in our study and previous trials, we can see that the AEs of apatinib are manageable, based on physician awareness and patient education.

This study offers first-hand efficacy and safety data of apatinib in the real world, which are informative for physicians and patients. Secondly, exploratory analysis provides several clues for selection of patients who are more likely to benefit from apatinib. Thirdly, safety analysis of this study helps gain better knowledge and familiarization with possible side effects and how to deal with them. However, this study was subject to limitations of its retrospective observational methodology, including potential missing data, possible information bias, small sample size and lack of control group. Moreover, quality of life was not formerly assessed, which could have provided more comprehensive information on apatinib toxicities. Furthermore, potential crowds remained to be crystallized.

Taken together, the efficacy and safety profiles of apatinib in this study were similar to previous clinic trials. Heavily pretreated advanced gastric cancer patients can tolerate and benefit from apatinib therapy, which makes apatinib therapy a promising option. And the specific application strategies need further exploration.

## Methods

### Patients

This is a retrospective real world study approved by the ethics committee of Chinese PLA General Hospital (PLAGH). Patients with advanced gastric adenocarcinoma or adenocarcinoma of the gastroesophageal junction who had progressed or relapsed after undergoing at least two lines of systemic therapy in accordance with the recommendations and guidelines of NCCN (National Comprehensive Cancer Network) in Oncology Department of the Chinese PLAGH between January 2015 and February 2017 were included. Data were obtained from patients’ medical history. Demographic and clinical characteristics and previous treatment were evaluated in all patients.

### Treatment

Apatinib therapy was initiated from an oral administration dosage of 500 mg once a day, 4 weeks for a cycle, which could be adjusted according to patients’ actual performance status ranging from 750 mg to 250 mg once daily. The daily dosage could be decreased to 250 mg due to patients’ severe adverse events. The chemotherapy or targeted therapy combined with apatinib was hinged on physicians’ determination based on patients’ situations. Informed consent was reviewed and signed by the patients or their legal guardian before apatinib therapy.

Only patients who had finished at least one cycle apatinib therapy and evaluated the efficacy were included in this study.

### Efficacy and safety

The efficacy of apatinib was evaluated including progression-free survival (PFS), overall survival (OS), objective response rate (ORR), disease control rate (DCR). PFS was defined as time from initiation of apatinib to disease progression or death, whichever occurred first. OS was defined as the duration from the time of treatment initiation to the time of death of any cause or the last follow-up time. Tumor responses were evaluated by computed tomography (CT), magnetic resonance imaging (MRI), bone scan and physical examination every cycle until disease progression, categorized as complete response (CR), partial response (PR), stable disease (SD) and progressive disease (PD) which were confirmed by physicians according to Response Evaluation Criteria in Solid Tumors (RECIST) 1.1 criteria. ORR was estimated as the percentage of CRs and PRs. DCR was considered as the percentage of CRs, PRs, and SDs.

All adverse events (AEs) were reviewed and determined from patients’ medical history and laboratory examination results or from telephone follow-up according to the National Cancer Institute Common Terminology Criteria for Adverse Events version 4.0.

### Statistical analysis

PFS and OS and their corresponding 95% confidence intervals (CIs) were estimated by Kaplan-Meier method. The hazard ratios (HRs) and corresponding 95% confidence intervals (95% CIs) were estimated using the Cox’s proportional hazards regression model.

Quantitative variables are presented as median (range) or number of patients (percentage).

Exploratory analysis for potential factors to predict PFS of Apatinib was a two-step process, consisting of univariate analysis and multivariate analysis. Univariate analyses were performed with the Log-rank test. Factors included in the univariate analysis were age (<median = 58 vs. ≥58), prior gastrostomy (yes vs. no), liver metastasis (yes vs. no), peritoneal metastasis (yes vs. no), previous anti-Her2 therapy (yes vs. no), previous antiangiogenic therapy (yes vs. no) and combination therapy (apatinib only vs. combined with chemo or other targeted therapy). Multivariate analyses were performed with the Cox’s proportional hazards regression model based on results of the univariate analyses. All crude and adjusted HRs and 95% CIs were estimated. All statistical analyses were two sided. HR < 1 implied a lower risk of progression for patients. A statistical significance cutoff of p = 0.05 was used to retain the variables in the final model.

Responses and AEs were both aggregated in the form of frequency counts and percentages. The ORR and DCR analyses were based on frequency counts.

All the statistical analyses were performed using SPSS for Windows (version 21; IBM®, Armonk, NY).
